# Neighbourhood Influences on Children's Weight-related Behaviours and Body Mass Index

**DOI:** 10.3934/publichealth.2015.3.501

**Published:** 2015-08-31

**Authors:** Gabrielle L. Jenkin, Amber L. Pearson, Graham Bentham, Peter Day, Simon Kingham

**Affiliations:** 1 Department of Public Health, University of Otago, 23A Mein Street, Newtown, PO Box 7343, Wellington 6242, New Zealand; 2 Department of Geography, Michigan State University, 673 Auditorium Road, East Lansing, MI, 48823, USA; 3 School of Environmental Sciences, University of East Anglia, Norwich NR4 7TJ, United Kingdom.; 4 Department of Geography, GeoHealth Laboratory,University of Canterbury, Private Box 1400, Christchurch 8140, New Zealand.

**Keywords:** neighbourhood, obesity, BMI children, diet, physical activity

## Abstract

**Introduction:**

Neighbourhood contextual factors such as accessibility of food shops and green spaces are associated with adult bodyweight but not necessarily weight-related behaviours. Whether these associations are replicated amongst children is unknown.

**Aim:**

To understand which aspects of childrens' neighbourhoods are associated with unhealthy weight and weight-related behaviours.

**Methods:**

Individual-level data for children from the 2006/7 New Zealand Health Survey (of Body Mass Index (BMI), dietary indicators and socioeconomic variables) were linked with geographic level data on neighbourhood deprivation, rural/urban status, percentage of community engaged in active travel, access to green space, food shops and sports/leisure facilities. Logistic regression models were fitted for measures of BMI and weight-related behaviours; sugar sweetened beverage (SSB) consumption; fast-food consumption; and television viewing.

**Results:**

Increased community engagement in active transport was, counterintuitively, the only neighbourhood contextual factor associated with unhealthy weight amongst children. After adjustment for socioeconomic and environmental variables, greater access to green space appeared to have a protective effect on SSB consumption and neighbourhood deprivation was associated with all three unhealthy weight-related behaviours (SSB and fast-food consumption and television viewing).

**Conclusions:**

Although further research is needed, evidence from the current study suggests that a repertoire of health promotion interventions and policies to change unhealthy weight-related behaviours in high deprivation neighbourhoods may be required to address childhood obesity.

## 1. Introduction

New Zealand ranks 5^th^ of 33 OECD countries for childhood overweight and obesity combined[1] and recent data shows a significant increase in obesity amongst New Zealand children from 8% in 2008 to 10.7% in 2012.[2] Obese children are at increased risk of fractures, insulin resistance and psychological problems, and as they are more likely to become obese as adults there are implications for chronic disease and disability later in life[3],[4].

For adults and children alike, ultimately, physical inactivity and an unhealthy diet (high in sugar, fat or salt and low in fibre and micronutrients) are the key causes of excess weight gain.[5],[6] Individual choices regarding dietary and physical activity are often argued to be responsible for excess weight gain. However, it is increasingly accepted that such choices do not occur in a vacuum but in the context of what has been described as the ‘obesogenic’ environment: “the sum of the influences that the surroundings, opportunities or conditions of life have on promoting obesity in individuals and populations”.[7] Put simply, obesity has become a “normal response to an abnormal environment”.[8] The degree to which an environment, or more specifically a neighbourhood, is obesogenic, is likely to have an important bearing on the physical activity and dietary options available to residents.

The effects of the obesogenic environment may be much more significant for children because they have limited control over where and for how long they spend their time. The residential neighbourhoods of children are therefore likely to be especially important due to their limited geographic mobility. Although not consistently, neighbourhoods and neighbourhood characteristics have been found to be associated with differences in Body Mass Index (BMI) amongst adults.[9]–[12] For children, differences in BMI have been reported by neighbourhood socioeconomic status (higher rates of obesity are associated with increased deprivation) and by rural and urban status, with most studies reporting higher prevalence of unhealthy weight in rural areas[13]–[15], although the reverse has been found to be the case in some countries (China)[16]. However, strong empirical evidence of an association between BMI and most other neighbourhood characteristics is not yet available.[17]

Part of the explanation for neighbourhood variations in childrens' bodyweight might be found in features of the environment that can influence individual dietary and physical activity behaviours. As increased consumption of fast food[18] and sugar sweetened beverages (SSBs)[19] have been dentified as ‘probable’ causes of weight gain,[5],[6] at the neighbourhhod level, one might expect that greater access to food shops selling these products would be linked with their increased consumption. Similarly, there are features of the environment that can promote physical inactivity. These include urban design that prioritises motor vehicle transport over ‘active’ travel (such as walking or cycling),[20],[21] and access to recreational facilities[22] and public recreational spaces (and for children especially, parks and playgrounds[17]). For children, access to quality public recreational spaces may be particularly important for encouraging physical activity as it provides a free alternative to other common childrens' pasttimes like television viewing, which has been identified as ‘probable’ cause of weight gain amongst adults[6] and children.[23]–[25]

Of the few studies focused on children that have examined neighbourhood characteristics (location of food shops and public recreation spaces), dietary behaviours (consumption of fast food or SSBs) and physical activity related behaviours (typically the sedentary behaviours of television viewing or other screentime activities), the results have been mixed.[17] For instance, an Australian study found that living near to good quality public open spaces decreased the time children spent in the sedentary behaviours of television viewing and computer/e-games.[26] Another Australian study examining the links between neighbourhood characteristics, including access to quality recreational spaces and television viewing amongst children and adolescents, reported that access to good sporting facilities (as measured by parental perspectives) was associated with less television viewing.[27] Television viewing itself is also linked with the consumption of unhealthy food by children because it increases their exposure to unhealthy food marketing.[23]–[25] This was evident in a Canadian study where increased time spent in front of screens was linked with increased consumption of SSBs in preschool children.[26]

Yet some of the associations found in such studies have been counterintuitive. One study, for example, found that living within 1km of food shops was associated with less SSB consumption by children.[28] Inconsistencies in the results of studies investigating associations between neighbourhood characteristics and unhealthy weight-related behaviours amongst children are therefore evident. It has been suggested that such inconsistencies may be due to a number of methodological limitations including, the cross sectional design of the research, variation in the definition of variables and the geographic boundary of neighbourhoods, and if and how potential confounders such as socioeconomic status are statistically dealt with in the analysis.[17] Given this background, and as there has not yet been any similar research on New Zealand children, this study aimed to understand which aspects of childrens' neighbourhoods are associated with unhealthy weight and unhealthy weight-related behaviours in a New Zealand national sample.

## 2. Methods

The study was based on analyses (conducted in 2013) using individual-level data from the 2006/7 New Zealand Health Survey (NZHS) on obesity, diet and physical activity[29] linked to geographic information from other sources on potentially aetiologically-relevant environmental factors, based on the child's residential address at the time of the survey. This research did not require IRB approval. However, access to the data used in this study was provided by Statistics New Zealand under conditions designed to give effect to the security and confidentiality provisions of the Statistics Act of 1975. The results presented in this study are the work of the authors, not Statistics New Zealand.

### 2.1. Healthy and unhealthy weight-related behaviour data

The 2006/07 NZHS was conducted from October 2006 to November 2007. Data were collected for 4,921 children aged 14 years and younger (response rate of 71%). The NZHS is a key component of national health monitoring by the New Zealand Ministry of Health and is designed to be a nationally-representative sample of New Zealand children. This survey used a multi-stage, stratified, probability proportionate to size sample design, with increased sampling of some ethnic groups. A full description of the sampling design is available online.[30] Each selected child's primary caregiver (biological parent 90% of the time) was invited to participate in the child questionnaire. Height and weight (for children over 2 years) were taken using professional weighing scales, a portable stadiometer and an anthropometric measuring tape. Our sample was limited to the 4175 children (85% of original sample) for whom these measurements were available (those aged 2 and older).

For our research purposes, the NZHS health indicators and health behavioural end-points of interest were: 1) overweight; 2) obesity; 3) overweight or obesity (all defined using the international BMI classification for children); 4) sugar sweetened beverage consumption (three or more times per week); 5) TV viewing (two or more hours per day); and 6) fast-food consumption (three or more times per week). The choice of categories used were dictated by the survey itself.[31] These variables were then assigned a binary (1/0) value and were used as the outcomes of interest in statistical analyses.

### 2.2. Neighbourhood environmental characteristics data and potential confounder data

Drawing on the framework outlined by Sallis *et al*[22] the environmental characteristics in this study were access,measured in distance (rather than density as we expected that children would be more influenced by distance due to their limited mobility) to ‘foodshop’ (supermarkets, convenience stores and fast food retailers) outlets [32], proportion of green space in neighbourhood[33], area-level deprivation[34], access to gym/pools[35], urban/rural classification[36] and percentage of the adult resident population using active transport to work (used to indicate whether there is a community-level contextual norm of engaging in physical activity).[37] In addition to considering area-level deprivation as a neighbourhood-level exposure, this variable was considered as a potential confounder of the associations between the other neighbourhood-level exposures and BMI and weight-related behaviour outcomes. The environmental variables, the data source, their measurement and descriptive statistics are outlined in Table 1.

The number of variables was limited and all continuous variables were converted to quintiles (1 = low and 5 = high) to conform with Ministry of Health confidentiality requirements. The selected neighbourhood variables were then linked, by the Ministry of Health, to the individual-level NZHS responses by the residential address at the time of the survey (addresses were removed for anonymity prior to analyses). Some variables were measured at the meshblock level (average 2006 population ∼ 100), which is the smallest unit of aggregation in New Zealand. Others were measured at the census area unit (CAU) level (average 2006 population ∼2500), which is the next largeest unit of aggregation and usefully approximates a neighbourhood in urban settings.

### 2.3. Individual-level potential confounder data

The NZHS also provides data at the individual-level on potential confounders including age (categorical in one year age bands), sex, ethnicity (Maori/Asian/Pacific/Other), parents' income (ordinal), parents' highest educational qualification, parents' employment status (working in paid employment/not in paid employment and looking for a job/not in paid employment and not looking for a job),, household composition, and the family's homeownership status (renting or owned).

### 2.4. Statistical analyses

Separate logistic regression models were fitted for the six binary dependent variables: 1) overweight; 2) obesity; 3) overweight+obesity; 4) sugar sweetened beverage consumption three or more times per week; 5) TV viewing two or more hours per day; and 6) fast-food consumption three or more times per week. Each model was first fitted unadjusted (i.e., each neighbourhood environmental factor one at a time for each of the dependent variables). Next, each model was fitted adjusted for individual-level confounders. Last, models were fitted for each dependent variable including all environmental factors as independent variables and adjusted for individual-level and area-level covariates. We included the independent environmental characteristics of interest (quintiles) as continuous variables to provide tests of trend and as discrete categories for which adjusted Odds Ratios (ORs) and 95% confidence intervals were calculated. Ethnicity was treated as a categorical variable (Maori, Asian, Pacific and ‘other’ (which includes European). All models were fitted using Stata v11 (College Station, TX, USA) with adjustment for the complex sample design of the NZHS, which produced cluster robust estimates. The analysis was based on all respondents who had complete data on all the variables.

**Table 1.** Sources and descriptions of neighbourhood environmental characteristics data

Characteristic	Description	Source (Year)	Descriptive statistics*
Urban/rural category	CAU ranking 1 to 4,where 1 = most urban	Statistics New Zealand (2006)	Min = 1, 25th percentile = 1, Mean = 1.4, Median = 1, 75th percentile = 1, Max = 4
Area-level deprivation (NZDep)	NZDep 2006 quintiles formeshblocks, where 1 = least deprived	Salmond (2006)[34]	Min = 1, 25th percentile = 2, Mean = 3.2, Median = 3, 75th percentile = 5, Max = 5
Accessibility of useable greenspace	Proportion of meshblockconsisting of useable greenspace, as quntiles where 5 = best access	Richardson (2005)[33]	Min = 1, 25th percentile = 2, Mean = 2.9, Median = 3, 75th percentile = 4, Max = 5
Accessibility of food outlets	Distance from meshblockpopulation-weighted centroidto nearest outlet (supermarkets,fast-food outlets, conveniencestores), as quintiles where 1 = nearest	Territorial Authorities (2005)[32]	Min = 1, 25th percentile = 2, Mean = 2.8, Median = 3, 75th percentile = 4, Max = 5
Accessibility of gym/poolfacilities	Distance from population-weighted centroid of meshblock to nearest gym, pool, karate, recreation centre, as quintiles where 1 = nearest (excludes biking/hiking trails)	ACC Pool Safety (2005)[35]	Min = 1, 25th percentile = 2, Mean = 2.8, Median = 3, 75th percentile = 4, Max = 5
Percentage active transport to work	Proportion of CAU adult residents who walk, bus or cycle to work, as quintiles, where 1 = least	Statistics New Zealand (2006)	Min = 1, 25th percentile = 2, Mean = 3, Median = 3, 75th percentile = 4, Max = 5

*Calculated only for areas where health survey participants resided.

## 3. Results

Table 2 shows that the majority of children in the sample were not classified as either overweight or obese (67%). The percentage of overweight/obese children was similar between males and females. Younger respondents had lower levels of obesity, but overweight status was fairly consistent across all age groups. Among ethnic groups (for which respondents could self-identify with more than one group), those identifying as Pacific had the highest percentages of both overweight (32%) and obese (24%), followed by those identifying as Māori. The children identifying as Asian had the lowest levels of obesity (6%), followed by those identifying as European (7%).

**Table 2.** Descriptive statistics for New Zealand children in the sample

	n	Overweight (%)	Obese (%)
**Total**	4175	930 (22)	439 (11)
**Age**			
2-4	958	219 (23)	84 (9)
5-8	1200	253 (21)	123 (10)
9-11	922	212 (23)	116 (13)
12-14	1095	246 (23)	116 (11)
**Sex**			
Male	2209	504 (23)	214 (10)
Female	1966	426 (22)	225 (12)
Ethnicity*			
Maori	1653	427 (26)	209 (13)
Pacific	670	211 (32)	160 (24)
European	2450	530 (22)	175 (7)
Asian	618	94 (15)	35 (6)
Other	41	5 (12)	4 (10)

*These are not exclusive categories, respondents could choose more than one self-identified ethnicity category

The results of our regression analyses, where the ORs represent tests of overall trends, indicate that overweight and overweight+obesity outcome categories exhibited significant associations with neighbourhood deprivation and access to foodshops in the unadjusted models (Model 1, Table 3). However, these associations attenuated after adjustment for both individual-level confounders and the other environmental characteristics (Model 3). The only environmental factor with a persistent effect across the models was percentage of the community engaging in active travel. In other words, residing in neighbourhoods with higher percentages of active transport was found to be associated with being overweight or obese.

The results of our unadjusted regression analyses for unhealthy weight-related behaviours indicate that living at greater distances from foodshops was significantly associated with lower fast food consumption (OR = 0.82, *p* < 0.0001). Greater access to greenspace was significantly associated with lower SSB consumption(OR = 0.93, *p* = 0.040), and neighbourhood deprivation was significantly positively associated with all three behaviours (Table 4). These trends remained significant after adjustment for socio-demographic variables (Model 2). However, in the fully adjusted model (Model 3), only neighbourhood deprivation was associated with all three unhealthy weight-related behaviours. Also, access to greenspace continued to exhibit an apparent protective effect on SSB consumption (OR = 0.91, *p* = 0.043).

**Table 3.** Associations (tests of trend) between overweight, obesity, overweight+obesity and environmental factors

	MODEL 1 *(run separately for each environmental factor)* Unadjusted			MODEL 2 *(run separately for each environmental factor)*Adjusted individual factors			MODEL 3 *(all environmental factors included)*Adjusted individual factors and environmental factors		
	OR	95% CI	p-value	OR	95%CI.	p-value	OR	95%CI.	p-value
**Overweight**									
Urban/rural	1.05	0.92, 1.20	0.460	1.09	0.94, 1.26	0.237	1.12	0.93, 1.36	0.235
NZdep	**1.21**	**1.12, 1.31**	**< 0.0001**	**1.10**	**1.00, 1.22**	**0.046**	1.10	0.99, 1.23	0.090
Greenspace	0.94	0.87, 1.01	0.112	0.94	0.86, 1.02	0.115	.094	0.86, 1.04	0.233
Foodshop	**0.91**	**0.84, 0.99**	**0.029**	0.93	0.85, 1.02	0.134	0.98	0.87, 1.11	0.769
Gym/pool	1.02	0.94, 1.10	0.599	1.00	0.91, 1.10	0.998	1.05	0.92, 1.19	0.467
Active travel	**1.08**	**1.01, 1.17**	**0.036**	**1.13**	**1.04, 1.23**	**0.003**	**1.20**	**1.09, 1.32**	**< 0.0001**
**Obesity**									
Urban/rural	0.78	0.63, 0.97	0.026	0.91	0.70, 1.18	0.467	0.89	0.65, 1.22	0.460
NZdep	1.41	1.25, 1.58	< 0.0001	1.07	0.90, 1.27	0.436	1.14	0.97, 1.33	0.110
Greenspace	1.05	0.95, 1.16	0.310	1.03	0.91, 1.17	0.668	1.01	0.88, 1.16	0.897
Foodshop	0.85	0.76, 0.94	0.002	0.96	0.85, 1.08	0.460	1.09	0.93, 1.29	0.292
Gym/pool	0.94	0.85, 1.03	0.190	0.96	0.85, 1.09	0.535	1.08	0.93, 1.25	0.330
Active travel	**1.15**	**1.06, 1.26**	**0.001**	**1.19**	**1.08, 1.32**	**0.001**	**1.23**	**1.08, 1.40**	**0.001**
**Overweight+obesity**									
Urban/rural	0.98	0.86, 1.11	0.764	1.06	0.92, 1.21	0.423	0.84	0.91, 1.28	0.402
NZdep	**1.26**	**1.17, 1.35**	**< 0.0001**	1.09	1.00, 1.19	0.059	1.10	1.00, 1.22	0.054
Greenspace	0.97	0.90, 1.04	0.403	0.96	0.89, 1.03	0.256	0.96	0.88, 1.04	0.312
Foodshop	**0.89**	**0.83, 0.96**	**0.003**	0.94	0.85, 1.02	0.119	1.01	0.90, 1.12	0.910
Gym/pool	1.00	0.93, 1.07	0.927	0.99	0.91, 1.07	0.791	1.05	0.94, 1.17	0.373
Active travel	**1.11**	**1.03, 1.18**	**0.003**	**1.15**	**1.07, 1.23**	**< 0.0001**	**1.21**	**1.11, 1.32**	**< 0.0001**

Bold *p* < 0. 05

**Table 4.** Associations (tests of trend) between unhealthy weight-related behaviours and environmental factors

	MODEL 1 *(run separately for each environmental factor)* Unadjusted			MODEL 2 *(run separately for each environmental factor)* Adjusted individual factors			MODEL 3 *(all environmental factors included)* Adjusted individual factors and environmental factors		
	OR	95% CI	p-value	OR	95%CI.	p-value	OR	95%CI.	p-value
**TV viewing +2hr/week**									
Urban/rural	1.08	0.97, 1.20	0.177	1.02	0.90, 1.14	0.811	0.96	0.81, 1.14	0.645
NZdep	**1.29**	**1.21, 1.38**	**< 0.0001**	**1.23**	**1.12, 1.34**	**<0.0001**	**1.24**	**1.13, 1.37**	**< 0.0001**
Greenspace	0.95	0.88, 1.03	0.200	0.97	0.89, 1.06	0.471	0.94	0.85, 1.04	0.224
Foodshop	0.95	0.88, 1.03	0.193	0.95	0.87, 1.04	0.268	1.04	0.93, 1.18	0.484
Gym/pool	1.05	0.97, 1.13	0.222	1.00	0.92, 1.09	0.940	1.05	0.93, 1.19	0.455
Active travel	1.04	0.96, 1.12	0.363	1.08	0.99, 1.17	0.090	1.09	0.98, 1.21	0.098
**Fast-food 3+ times/week**									
Urban/rural	0.85	0.70, 1.04	0.119	0.88	0.70, 1.11	0.293	0.92	0.70, 1.21	0.541
NZdep	**1.46**	**1.29, 1.65**	**< 0.0001**	**1.22**	**1.06, 1.41**	**0.006**	**1.19**	**1.02, 1.39**	**0.030**
Greenspace	0.99	0.89, 1.12	0.930	1.00	0.87, 1.14	0.964	0.98	0.83, 1.15	0.791
Foodshop	**0.82**	**0.73, 0.91**	**< 0.0001**	**0.86**	**0.76, 0.98**	**0.027**	0.96	0.78, 1.17	0.673
Gym/pool	0.95	0.85, 1.06	0.348	0.94	0.82, 1.07	0.325	0.99	0.82, 1.21	0.948
Active travel	1.04	0.94, 1.14	0.487	1.05	0.93, 1.18	0.453	1.01	0.85, 1.19	0.950
**SSB 3+ times/week**									
Urban/rural	1.03	0.91, 1.16	0.669	1.10	0.96, 1.27	0.155	0.97	0.81, 1.15	0.690
NZdep	**1.18**	**1.09, 1.26**	**< 0.0001**	**1.10**	**1.00, 1.20**	**0.044**	**1.14**	**1.03, 1.26**	**0.009**
Greenspace	**0.93**	**0.86, 1.00**	**0.040**	**0.98**	**0.82, 1.05**	**0.005**	**0.91**	**0.83, 1.00**	**0.043**
Foodshop	0.98	0.91, 1.06	0.609	1.08	0.99, 1.18	0.085	1.09	0.96, 1.23	0.171
Gym/pool	1.03	0.95, 1.12	0.495	**1.10**	**1.00, 1.19**	**0.039**	1.03	0.92, 1.16	0.609
Active travel	0.97	0.91, 1.04	0.431	0.94	0.87, 1.02	0.150	1.00	0.91, 1.10	0.974

Note: SSB = Sugar-sweetened beverage; Bold *p* < 0. 05

## 4. Discussion

To elucidate which contextual aspects of New Zealand childrens' residential environments were associated with unhealthy bodyweight, we examined urban/rural differences, neighbourhood deprivation, access to green space, recreational facilities (gyms and pools) access to food shops (healthy and unhealthy), and community engagement in active travel. Community engagement in active travel was the only neighbourhood contextual factor associated with higher BMI amongst children.

In examining aspects of residential environments associated with unhealthy weight-related behaviors, we found that a greater proportion of neighbourhood green space was associated with lower SSB consumption (although this could be spurious). Additionally, we found that neighbourhood deprivation was significantly associated with all three obesity related behaviours (SSB and fast food consumption and television viewing). Importantly, these associations between unhealthy weight-related health behaviours of children and neighbourhood contextual factors occurred independent of a variety of covariates.

If we consider the unexpected positive association between community levels of active travel and bodyweight amongst children, the consistency and strength of the associations with overweight and obesity suggests that this is not a chance result. However, the observed association is the opposite to what might be expected. We can only speculate that neighbourhoods with high levels of active travel to work for adults may also have other unmeasured characteristics which actually influence child overweight and obesity. Another possible explanation for this curious finding may relate to the potential heterogeneous nature of neighbourhoods with high levels of active transport. These neighbourhoods tend to be urban settings[38] and also tend to be more deprived, as our research has shown. It is possible that these neighbourhoods are made up of a heterogenous population consisting of families (with overweight or obese children) and young people (under 25 years) who cannot afford to live in a more affluent neighbourhood and who tend to actively commute to work, as other research has shown.[38] This potential explanation was outside the scope of the current research, however, it warrants further exploration.

Alternatively, active transport patterns of adults in the community may not be mirrored amongst children. Unfortunately, the absence of physical activity data for children in our sample makes this impossible to determine. Further, active transport rates were consistently fairly low[38] and this may affect the validity of these findings.

We are unclear as to why there appears to be a link between access to green space and SSB consumption although a link between access to recreational spaces (good sporting facilities) and less television viewing has been reported.[27] One possible reason for this may be that childrens' access to greenspace may displace screentime activity, particularly television viewing, which has been found to be associated with SSB consumption by children.[26]

However, our key finding, living in a socioeconomically deprived neighbourhood is associated with unhealthy weight-related behaviours (all three of them), has been reported consistently, according to a recently systematic review review of obesogenic dietary behaviours and environmental factors.[9] Other evidence from the same review[9] and elsewhere[39] suggests, that associations between the environment and weight status are more consistent than those found between the environment and dietary behaviours. Our findings appear to contradict this, as we found only one environmental variable associated with BMI in children.

### 4.1. Strengths and limitations

The health behaviour measures used in the current research, being based on caregiver self-report, may be subject to some measurement errors. Caregivers may not know how much time their children spend watching television and/or may not know or be reluctant to disclose the frequency at which their children consume SSBs or fast foods. As noted above, the measure of foodshop is also limited in this study as it does not distinguish between type of food shops, in particular between access to fast food versus supermarkets, which are known to be variously associated with BMI.[40] We were also limited in the number of environmental measures that could be used in this research due to confidentiality requirements of the Ministry of Health. Future research could therefore examine a wider range of environmental variables, the density of foodshops in the area and for school aged children especially, environmental exposures around schools. We also note that as there was no adjustment for multiple testing in this study, the association between neighbourhood green space and SSB consumption may be spurious. Further research is needed to clarify this.

However, this research has a number of strengths. It is one of the few studies that have recenty emerged[39],[41]–[44] to examine neighbourhood contextual influences on children's bodyweight, and it is novel in being the first such study to examine the situation for New Zealand children. Further strengths of the current study are its large and nationally reporesentative sample, the high response rate and the objective measurement of bodyweight.

### 4.2. Implications

Although additional research is required to address the shortcomings in this research and to further our understanding of the role of neighbourhood contextual factors on childrens' health related behaviours, the associations between living in a socioeconomically deprived neighbourhood and unhealthy weight related behaviours suggest a number of potential opportunities for health promotion. In the first instance, interventions to change unhealthy weight related behaviours could be targeted to high deprivation neighbourhoods and be implemented at the family, community or school levels. Such interventions need not assume a knowledge deficit model and focus on educating the families resident in the community, although this is one option, but could take a wider community development approach and consider environmental interventions. These could include the increased provision and promotion of public water fountains (to counteract the competition from SSBs), and the provision of community or school gardens to promote, normalise and increase the availability of healthy produce in the area. At the wider policy level, limiting the placement of fast food stores in high deprivation neighbourhoods is an option for improving the balance between healthy and unhealthy food supplies. Health promotion efforts to help get children away from the television might be geared towards improving opportunities for children's physical activity in high deprivation neighbourhoods. This might require assessing and addressing safety concerns in the area and promoting active transport to and from school. Food promotion on television itself should also be addressed.

## 5. Conclusion

Although further research is required, this is one of the few studies internationally to examine associations between the neighbourhood environment contextual factors and bodyweight amongst children, and the first of its kind in New Zealand. Although some of the findings are surprising, the key associations reported, between neighbourhood deprivation and unhealthy weight related behaviours are consistent with research findings internationally. We can take from this, that addressing geographic variations in child obesity is likely to require a repertoire of health promotion interventions and policies aimed at changing unhealthy weight related behaviours in high deprivation neighbourhoods.
